# In vitro bioactivity profile-driven discovery of drug synergies in hepatocellular carcinoma

**DOI:** 10.1186/s12967-026-08713-x

**Published:** 2026-07-28

**Authors:** Tuan Xu, Miao Xu, Deborah K. Ngan, Tongan Zhao, Catherine Z. Chen, Wei Zheng, Ruili Huang

**Affiliations:** Division of Pre-clinical Innovation, National Center for Advancing Translational Sciences (NCATS), National Institutes of Health (NIH), Rockville, MD 20850 USA

**Keywords:** Hepatocellular carcinoma, Drug combination, Synergy, Phenotypic profiling, Bioactivity signature, Context dependence

## Abstract

**Background:**

Combination therapy is a central strategy to overcome drug resistance in hepatocellular carcinoma (HCC), yet systematic identification of synergistic combinations is constrained by the combinatorial search space.

**Methods:**

We developed a data-driven workflow to integrate two orthogonal single-agent resources for 244 drugs, including cell viability profiles across 11 liver cancer cell lines and bioactivity signatures across 1,925 biochemical and cell-based assays.

**Results:**

We calculated the correlation of the bioactivity profiles between each drug pair yielding 821 high-confidence combinations involving 190 unique drugs, from which eight anchors and a 125-drug library were selected by frequency-guided prioritization. Fixed-concentration screening of 992 anchor-library combinations in the Hep 3B2.1-7 cell line yielded 89 combinations with potency enhancement. Panobinostat showed the largest potency gains when combined with briciclib or thiocolchicine (69.5-fold and 81.1-fold lower IC₅₀, respectively). Matrix-based screening of these two representative combinations across 11 cell lines revealed pronounced concentration- and context-dependence. At optimal dose pairs, strong synergy was observed in Hep 3B2.1-7 (Zero Interaction Potency (ZIP) = 40.80 ± 4.22 with 58.53 ± 6.54% inhibition), whereas antagonism was observed in some cellular backgrounds.

**Conclusion:**

This label-free integration strategy reduces the experimental burden for hypothesis-driven combination discovery and provides context-specific preclinical leads for follow-up evaluation in HCC.

**Supplementary Information:**

The online version contains supplementary material available at https://doi.org/10.1186/s12967-026-08713-x.

## Introduction

Hepatocellular carcinoma (HCC), the predominant subtype of primary liver cancer (75–85% of cases), remains a leading cause of cancer mortality worldwide [1]. Based on GLOBOCAN 2020 estimates, International Agency for Research on Cancer (IARC) projections predict a 55% increase in new liver cancer cases from 2020 to 2040, reaching approximately 1.4 million by 2040 [2]. This projected rise underscores the need for more effective preventive and therapeutic strategies. Clinically, HCC typically arises in the setting of chronic liver disease driven by diverse etiologies, including chronic hepatitis B virus (HBV) and hepatitis C virus (HCV) infection, aflatoxin exposure, heavy alcohol consumption, excess body weight, type 2 diabetes, and smoking [1]. For example, network-based analyses have also linked aflatoxin B1 exposure to oncogenic signaling in HCC, including predicted interactions with AKT serine/threonine kinase 1 (AKT1) associated with metastasis-related pathways [3]. Consistent with this etiologic diversity, HCC initiation and progression are associated with genomic alterations affecting tumor suppression and cell-cycle control (e.g., inactivating *TP53* mutations), telomere maintenance (e.g., telomerase reverse transcriptase (*TERT*) promoter mutations), oncogenic signaling (e.g., activating *CTNNB1*/β-catenin mutations), chromatin remodeling and other epigenetic regulators (e.g., AT-rich interaction domain 1 A (*ARID1A*) mutations), as well as *MAPK* and *mTOR* pathways and oxidative stress responses [4, 5]. This heterogeneity complicates durable disease control in advanced HCC [6], where resistance frequently emerges despite available systemic therapies such as sorafenib [7].

Combination therapy, the concurrent or sequential administration of agents with complementary or distinct mechanisms of action, is designed to enhance antitumor efficacy, delay or prevent resistance, and mitigate dose-limiting toxicities associated with high-dose monotherapy [8, 9]. This strategy has delivered clinical benefit across diverse malignancies, including rituximab plus bendamustine in lymphoma [10], and trastuzumab plus pertuzumab in HER2-positive breast cancer [11]. In HCC, combination regimens have further advanced clinical outcomes in selected settings. The IMbrave150 trial demonstrated that atezolizumab plus bevacizumab extended median overall survival from 13.4 to 19.2 months compared with sorafenib monotherapy [12]. The COSMIC-312 trial showed improved progression-free survival with cabozantinib plus atezolizumab (6.8 months) compared with sorafenib (4.2 months) [13], while the HIMALAYA study established tremelimumab plus durvalumab with median overall survival of 16.4 versus 13.8 months with sorafenib [14]. However, benefit is not uniform across combinations in HCC. For example, in the LEAP-002 trial, lenvatinib plus pembrolizumab did not meet prespecified significance for improving overall survival and progression-free survival versus lenvatinib alone [15]. Collectively, these variable outcomes underscore the need for scalable frameworks that prioritize combinations for liver-cancer-relevant testing rather than relying on empirical selection.

High-throughput screening (HTS) and computational modeling offer complementary approaches for systematically identifying synergistic drug combinations. Quantitative HTS (qHTS) enables titration-based testing of thousands of compounds or combinations in miniaturized 384- or 1536-well formats, generating concentration-response curves directly from primary screens and supporting robust pharmacological profiling and downstream synergy analysis [16, 17]. In liver cancer, HTS platforms have yielded candidate combinations with translational potential. For example, the Liver Cancer Organoid Biobank (LICOB), comprising 65 patient-derived organoids spanning major liver cancer subtypes, was used to screen 76 anticancer agents and identified temsirolimus plus lenvatinib as a promising doublet that was subsequently validated in organoid and patient-derived xenograft models [18]. In the Hep 3B2.1-7 HCC cell line, screening of 7,051 bioactive compounds in combination with sorafenib identified PD198306 as a strong synergistic partner of sorafenib [19]. Despite these advances, unbiased large-scale HTS remains resource-intensive and samples only a small fraction of the combinatorial space. To prioritize experimentally testable combinations more efficiently, computational methods have been developed to predict drug synergy from large pharmacogenomic datasets. For example, supervised models trained on pan-cancer resources, including deep learning approaches (e.g., DeepSynergy and MTLSynergy) [20, 21], can leverage chemical descriptors, molecular profiles and prior screening data to predict interaction outcomes. However, the applicability of these models to disease-specific settings such as HCC is limited because labeled synergy datasets remain sparse or unavailable for many HCC-relevant contexts [22], predictions may lack mechanistic interpretability needed for hypothesis generation [23], and generalization across new chemical scaffolds, assay protocols, or disease contexts remains uncertain [20]. These constraints motivate complementary approaches that do not require large, context-matched synergy labels and remain interpretable for hypothesis generation.

To address these limitations, we sought to develop a complementary, label-free strategy that integrates multiple single-agent profiling modalities. Compounds that demonstrate concordant bioactivity profiles across diverse cellular contexts may reflect shared or coupled molecular mechanisms or pathways [24, 25]. Correlations between compound bioactivity signatures can serve as a hypotheses generation tool for prioritizing experimentally testable combinations. In this study, we integrated cell viability profiles across liver cancer models with a large, diverse panel of bioactivity signatures to prioritize candidate drug combinations. Prioritized drug combinations were then experimentally evaluated using a panel of liver cancer cell lines in dose-response format including full matrix analysis. This study aims to establish a label-free workflow for efficient identification and prioritization of drug combinations to facilitate anticancer therapy development.

## Materials and methods

### Cell lines and culture conditions

Eleven cell lines were used, including nine HCC lines (Hep 3B2.1-7, HuH-7, PLC/PRF/5, SNU-182, SNU-387, SNU-398, SNU-423, SNU-449, and SNU-475), and two non-HCC liver cancer cell lines (Hep G2, a hepatoblastoma-derived line [26]; and SK-HEP-1, an endothelial-like line frequently used as a liver cancer model [27]) to broaden phenotypic coverage beyond canonical HCC contexts. All cell lines were obtained from the American Type Culture Collection (ATCC, Manassas, VA, USA) and maintained at 37 °C in a humidified atmosphere containing 5% CO₂. Cells were passaged at 80–90% confluence using 0.25% trypsin-EDTA (Gibco, cat. no. 25200-056), and experiments were performed between passages 3 and 10. Cell line identity was authenticated by short tandem repeat (STR) profiling within 6 months of experimental use and matched ATCC reference profiles with > 90% concordance. Mycoplasma contamination was tested monthly using the MycoAlert Mycoplasma Detection Kit (Lonza, cat. no. LT07-418), and all cultures tested negative throughout the study. Cell line characteristics, including tissue origin and *TP53* status, are summarized in Supplementary Table S1.

### HTS data collection

Biological activity data for 244 small-molecule drugs were compiled from two in-house qHTS resources at the National Center for Advancing Translational Sciences (NCATS). The first dataset comprised phenotypic cell viability measurements across 11 liver cancer cell lines (9 HCC lines, Hep G2, and SK-HEP-1). The NCATS in-house collection of ~ 10 K bioactive compounds, including the NCATS Pharmaceutical Collection of ~ 3000 approved drugs [28], were screened against the 11 liver cancer cell lines in qHTS format (PubChem AID: 2202548). Selected actives were retested at 11 concentrations across the same cell lines to confirm their activity. These data were used subsequently for correlation analysis. Compound activity was quantified using a composite score: Score = − log₁₀(IC₅₀) × efficacy / 100, where IC₅₀ is the half-maximal inhibitory concentration (molar units) estimated by concentration-response curve fitting, and efficacy is the maximal response magnitude relative to dimethyl sulfoxide (DMSO) controls (set to 100%; no cell killing). Using this metric, compounds with both high potency (low IC₅₀) and strong efficacy (high maximal inhibition) were assigned higher scores and considered more active. For inactive or non-classifiable curves, IC₅₀ was capped at 100 µM and efficacy was set to 0 for the purpose of score calculation. Efficacy values exceeding 100% reflect variability in normalized readouts and curve fitting. The second dataset was derived from the NCATS in-house bioactivity signatures generated by screening bioactive compound libraries across 1,925 biochemical and cell-based assays spanning diverse targets and pathway readouts. Each compound was tested in concentration-response format and summarized using curve rank, a direction-sensitive score (− 9 to + 9) integrating potency, efficacy, and curve quality. Negative values indicate inhibitory/antagonist activity, positive values indicate agonist/activator activity, and zero indicates no significant response [29]. Compound annotations, assay sources, and raw measurements are provided in Supplementary Tables S2–S4.

### Correlation analysis

**Fig. 1:**
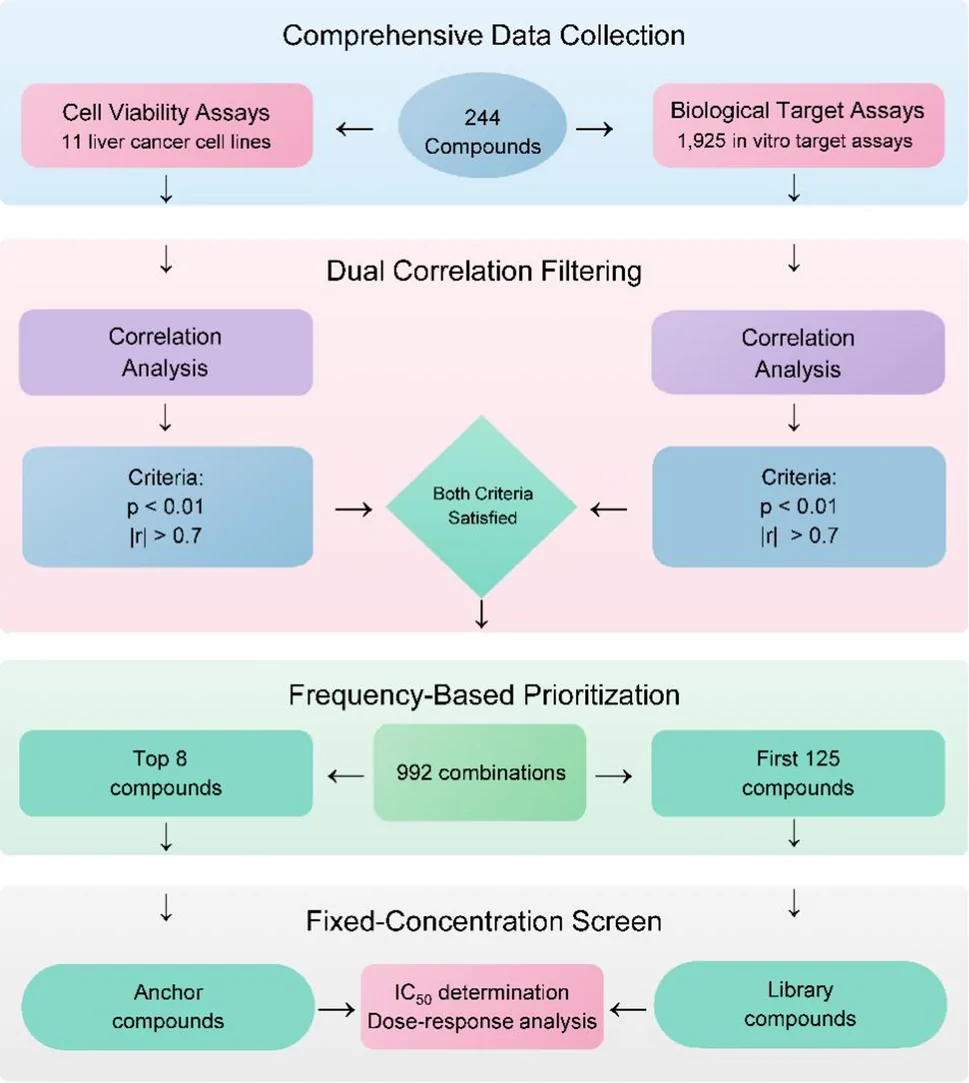
Dual-profile correlation framework for systematic combination prioritization. A selected library of 244 bioactive compounds was profiled in parallel using (i) cell-viability assays across 11 liver cancer cell lines and (ii) bioactivity signatures across 1,925 biochemical and cell-based assays. Pairwise Pearson correlations were computed independently in each dataset (bioactivity correlations were computed using overlapping non-missing assays for each compound pair), and compound pairs were retained only if they met dual criteria in both datasets (two-sided *p* < 0.01 and |*r*| > 0.7) with concordant correlation signs. Retained pairs were used to rank compounds by frequency of occurrence, enabling selection of eight anchor compounds and a 125-compound library (including anchors) for experimental testing. Anchor–library combinations were evaluated by fixed-anchor-concentration screening in Hep 3B2.1-7, and selected hits were subsequently characterized by dose–response matrix profiling across the full 11-cell-line panel

Pairwise Pearson correlation coefficients (r) were calculated for all compound pairs based on activity profiles across (i) 11 liver cancer cell lines using composite activity scores and (ii) 1,925 in vitro assays using curve rank values. Correlation coefficients and two-sided *p*-values were computed in R (v4.3.1) using cor() and cor.test() functions. For the cell viability profiles, all compound pairs were analyzed. For the bioactivity signatures, correlations were computed using only overlapping non-missing assays for each compound pair, and pairs with fewer than 3 overlapping assays were excluded from correlation analysis. Compound pairs with highly correlated (*|r*| > 0.7) activity profiles that were statistically significant (two-sided *p* < 0.01) in both datasets, and with concordant correlation signs between the two datasets, i.e., *r* > 0 or *r* < 0 in both datasets, were retained for downstream analyses. Compounds were then ranked by the frequency with which they appeared among retained pairs. Top-ranked compounds were designated as anchors, and additional top-ranked compounds (including anchors where applicable) were selected as the library for pairwise combination testing against each anchor. The correlation analysis workflow is presented in Fig. 1.

### Fixed-anchor-concentration combination screening (primary screen)

Primary combination screening was performed in the Hep 3B2.1-7 cell line in 1,536-well plates. For each anchor-library pair, the anchor compound was tested at a fixed concentration (0.5 µM) [19], while the library compound was tested across an 11-point, 3-fold serial dilution series starting from 45 µM. Compounds were dispensed using an Echo 550 acoustic liquid handler (Beckman Coulter Life Sciences), and plate layouts were randomized to mitigate positional effects. Cell viability was measured using CellTiter-Glo (Promega), and luminescence was read on a ViewLux plate reader (PerkinElmer). Potentiating hits were defined by potency-shift criteria using qHTS curve fits: (i) both the single-agent titration and the anchor-library titration produced active inhibitory curves (curve rank < − 1) and the combination IC₅₀ decreased by >6-fold relative to the single-agent, or (ii) the anchor-library titration produced an active inhibitory curve (curve rank < − 1) while the single-agent titration was inactive (curve rank ≥ − 1). Each condition was assayed in triplicate, and the screen was repeated across 2 − 5 independent biological experiments.

### Matrix-based combination screening (secondary screen)

Selected hits were further profiled across all 11 cell lines using an 11 × 11 dose-response matrix design. Each compound contributed 10 non-zero concentrations plus a 0 µM (DMSO) control, yielding 11 levels per compound and 121 (11 × 11) dose pairs per matrix. Cells were seeded into 1,536-well plates at optimized densities and incubated overnight. Compounds were dispensed using the Echo 550 and incubated for 4 h, and CellTiter-Glo viability was measured and normalized to DMSO controls. Each matrix experiment was performed in two independent biological replicates. Normalized matrices were analyzed using SynergyFinder (v3.6.0) [30] with default settings in R with four reference models: Zero Interaction Potency (ZIP), Highest Single Agent (HSA), Bliss Independence, and Loewe Additivity. In this study, ZIP was used as the primary synergy metric. Antagonistic dose pairs were defined as ZIP < − 10. Synergistic dose pairs were defined using a dual criterion of ZIP > 10 and viability reduction > 50%. Here, > 50% inhibition was required to prioritize synergistic dose pairs with meaningful phenotypic impact rather than numerically high synergy at near-baseline effects. All other pairs were classified as non-synergistic/non-antagonistic, including pairs with ZIP > 10 but ≤ 50% inhibition. For each dose pair, ZIP scores and viability reduction values were first averaged across the two biological replicates and then classified using the predefined thresholds.

### Statistical analysis

qHTS data from the primary screen data were processed using established protocols described previously [31], including IC₅₀ estimation, curve rank assignment, and efficacy (maximal response) calculation. Concentration-response curves were fit using four-parameter log-logistic regression (LL.4) with percent viability reduction as the response variable and log₁₀(concentration) as the independent variable using the drc package in R. Error bars denote standard deviation (SD) across independent biological experiments. The number of independent biological experiments is reported in the corresponding figure legends. Data visualization was performed using ggplot2 (v3.4.3) in R.

## Results

### Cell viability profiling reveals compound activity patterns across liver cancer cell lines

**Fig. 2:**
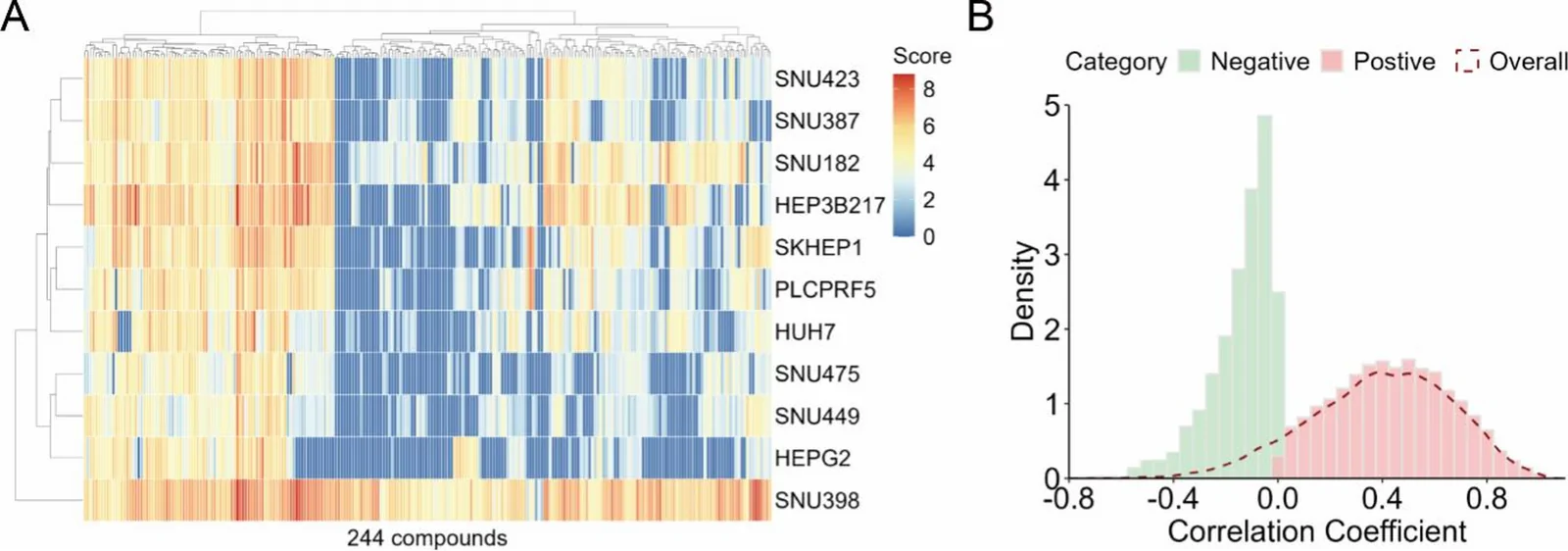
Cell viability profiling and compound-compound correlation analysis across liver cancer cell lines. (**A**) Heatmap of composite activity scores for 244 compounds across 11 liver cancer cell lines. The composite activity score was computed as Score = − log_10_(IC₅₀) × (efficacy/100), where IC₅₀ is the half-maximal inhibitory concentration (molar units) and efficacy is maximal viability reduction (%) relative to DMSO controls. Rows (cell lines) and columns (compounds) were clustered by Euclidean distance using complete linkage. (**B**) Distribution of pairwise Pearson correlation coefficients (*r*) for compound activity profiles across the 11 cell lines (29,646 pairs). Positive correlations indicate concordant phenotypic response patterns across cell lines, whereas negative correlations indicate inverse patterns

A selected library of 244 small molecule drugs was profiled for viability-reducing activity across 11 liver cancer cell lines (Fig. 2A, and Supplementary Tables S1-S3). The panel included nine HCC lines spanning diverse *TP53* backgrounds, including a *TP53*-null cell (Hep 3B2.1-7), *TP53*-altered cells, and *TP53* wild-type cells (Supplementary Table S1). The compound library is predominantly comprised of approved drugs or well-characterized bioactive agents (*n* = 226) and a smaller set of compounds with limited annotation (*n* = 18) (Supplementary Table S2). Composite activity scores ranged from 0 (e.g., SP-141 in HuH-7) to 8.8 (echinomycin in SNU-398), with a median of 3.7 across all compound-cell line combinations (Supplementary Table S3). Higher composite scores indicate stronger viability-reducing activity driven by both potency and efficacy. Hierarchical clustering of the activity profiles identified two major compound clusters: 89 compounds with higher overall activity (mean score = 5.20) and 155 compounds with lower activity (mean score = 2.53) (Fig. 2A). Average composite activity score also differed across cell lines. SNU-398 exhibited the highest average activity (score = 6.09), whereas Hep G2 showed the lowest average activity (score = 2.18). Other cell lines ranged from 2.36 (SNU-475) to 4.32 (SNU-182) (Supplementary Table S3). Pairwise Pearson correlation of cell viability profiles across the 11 cell lines was calculated for 29,646 compound pairs, of which 26,946 (90.9%) were positively correlated (*r* > 0) and 2,700 (9.1%) were negatively correlated (*r* < 0) (Fig. 2B). Correlation coefficients ranged from − 0.76 (eribulin mesylate vs. lometrexol hydrate) to 1.0 (e.g., cabazitaxel vs. rigosertib), with median *r* = 0.44 among positive pairs and − 0.10 among negative pairs.

### Diverse bioactivity profiling provides complementary compound signatures

**Fig. 3:**
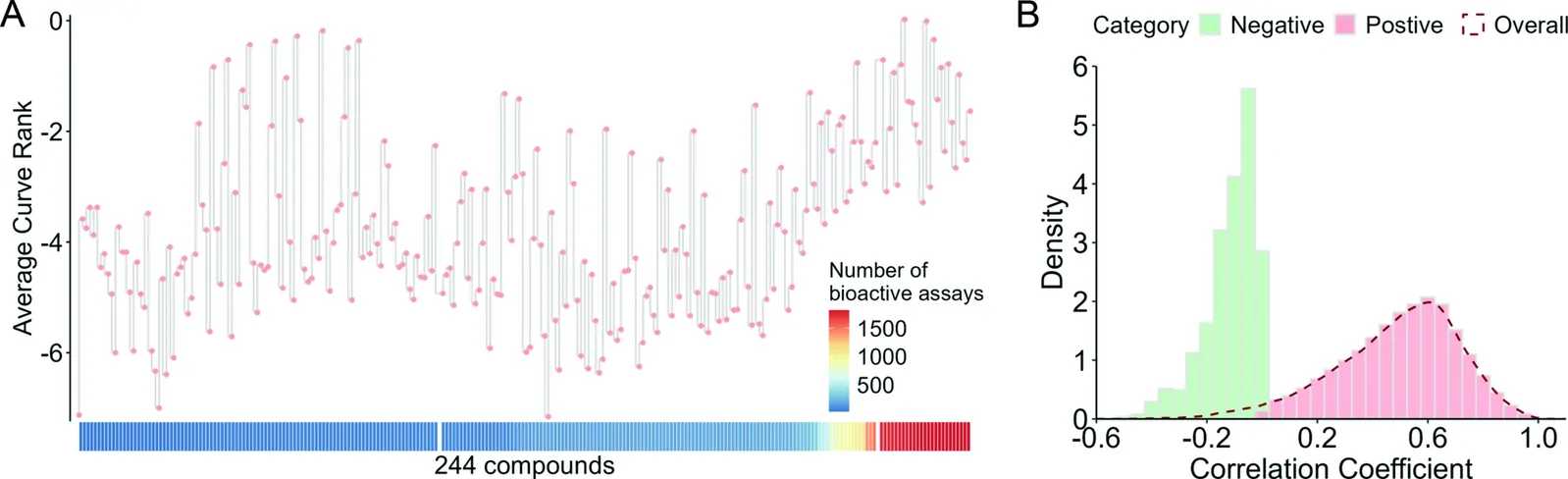
Bioactivity signature profiling and compound-compound correlation analysis across a biochemical and cell-based assay panel. (**A**) Distribution of compound-averaged curve rank values for 244 compounds across 1,925 assays. Curve rank is a numeric measure of compound activity (− 9 to + 9) integrating potency, efficacy, and curve quality, with negative values indicating inhibitory/antagonist activity, positive values indicating activation/agonism, and 0 indicating no significant activity. (**B**) Distribution of pairwise Pearson correlation coefficients (r) for compound bioactivity signatures (correlations computed using overlapping non-missing assays for each pair). Positive correlations indicate concordant bioactivity signatures, whereas negative correlations indicate inverse signatures

In addition to the liver cancer panel profile data, bioactivity profiles for the same 244 compounds screened across 1,925 biochemical and cell-based assays were retrieved from the NCATS in-house qHTS database (Fig. 3A, and Supplementary Table S4). This panel encompassed assays from multiple screening programs (Supplementary Table S4). Because compounds were not uniformly tested across all 1,925 assays, correlations were computed using only overlapping assays for each compound pair. The activity values were represented by curve rank ranging from − 9 (17.30% of observations) to + 9 (2.08%), with 0 indicating no significant activity (42.7%). Assay coverage per compound ranged from 24 (KP372-1) to 1,817 ((S)-apomorphine hydrochloride), with a median of 145 assays. Average curve rank per compound ranged from − 7.16 (sepantronium bromide, broadly inhibitory) to 0.02 (propylthiouracil, broadly inactive), with a median of − 4.04. Pairwise Pearson correlation of bioactivity signatures yielded 29,614 evaluable compound pairs (32 pairs excluded due to insufficient overlapping assay data). Among evaluable pairs, 28,602 (96.6%) were positively correlated (*r* > 0) and 1,012 (3.4%) were negatively correlated (*r* < 0) (Fig. 3B). For positively correlated pairs, *r* ranged from near-zero to 1.0 (vindesine sulfate salt vs. SB-1317) (median *r* = 0.53). For negatively correlated pairs, *r* ranged from − 0.72 (AZD-3147 vs. CGP-74514 A hydrochloride) to near-zero (median *r* = − 0.09).

### Dual filtering compresses the combinatorial space to high-confidence pairs

The 29,614 compound pairs with correlation coefficients calculated in both datasets were further filtered by correlation strength and significance. This dual-filtering approach reduced the combinatorial space from 29,614 evaluable pairs to 821 prioritized pairs (~ 36-fold) (Fig. 1, and Supplementary Table S5). The 821 prioritized pairs comprised 190 unique compounds (77.9% of the 244-compound library). Eight compounds (i.e., briciclib, DMEP, ELR-510444, epothilone D, MPT-0B098, nocodazole, TCS-JNK-5a, and thiocolchicine) were selected as anchors by correlation frequency-guided prioritization. Here, correlation frequency denotes the number of times a compound appears among the 821 prioritized pairs. The top 125 compounds (including the eight anchors) were used to construct anchor–library pairs by combining each anchor with the remaining 124 compounds (excluding self-pairs), yielding 992 combinations (8 × 124) for fixed-anchor-concentration primary screening. This design extends beyond the 821 strictly prioritized pairs by pairing each anchor with a prioritized library compound, increasing coverage while remaining experimentally tractable.

### Fixed-anchor-concentration screening identifies potentiating combinations in the Hep 3B2.1-7 cell line

**Fig. 4:**
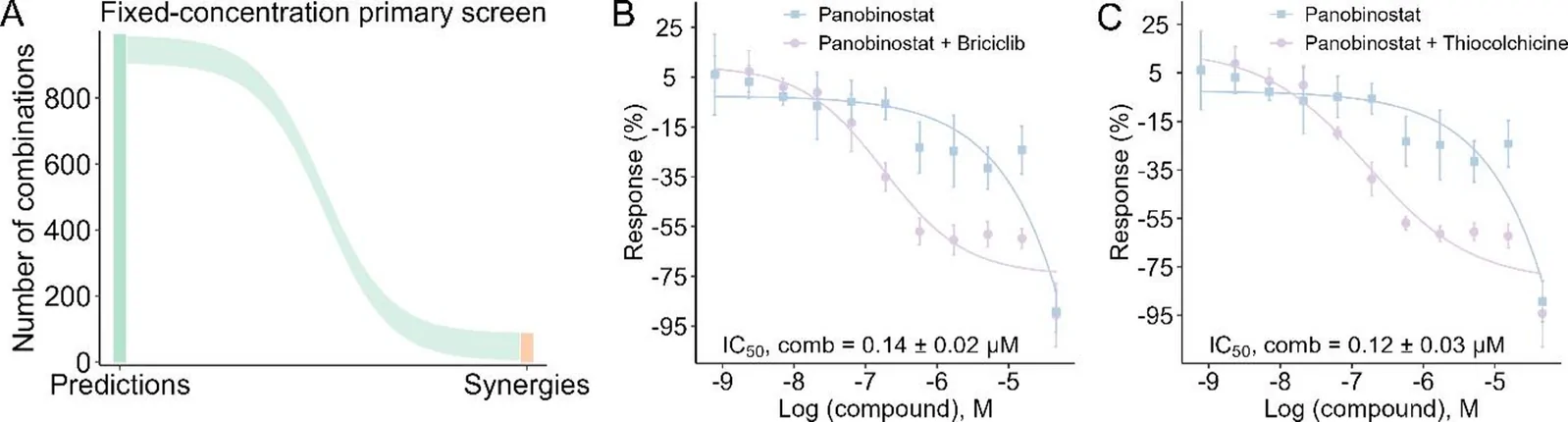
Fixed-anchor-concentration screening of prioritized combinations in the Hep 3B2.1-7 cell line. (**A**) Summary of potentiating hits identified from 992 anchor–library combinations screened in Hep 3B2.1-7. In the fixed-anchor-concentration format, each anchor was tested at 0.5 µM, while the partner compound was tested in an 11-point, 3-fold titration (starting at 45 µM). Potentiating hits were defined using predefined potency-enhancement criteria: (i) both the single-agent and combination titrations were active inhibitory curves (curve rank < − 1) and the combination IC₅₀ decreased by > 6-fold relative to single agent, or (ii) the combination titration was active (curve rank < − 1) whereas the single-agent titration was inactive (curve rank ≥ − 1). (B–C) Representative concentration–response curves illustrating potency enhancement for panobinostat in combination with briciclib (**B**) or thiocolchicine (**C**). Data are presented as mean ± standard deviation (SD), with error bars representing the SD of five independent experiments

Using a fixed-anchor-concentration design in the Hep 3B2.1-7 cell line, 992 anchor-library combinations were screened with each anchor at 0.5 µM and library compounds across an 11-point titration (Supplementary Table S6). At 0.5 µM, none of the anchor compounds showed viability reduction when tested alone (Supplementary Table S6). Among the 992 combinations, 89 (9.0%) met prespecified potency-enhancement criteria and were designated as potentiating hits (Fig. 4A). Representative concentration-response curves for panobinostat combined with briciclib or thiocolchicine illustrate marked potency improvement relative to single-agent panobinostat (Fig. 4B-C). In combination, panobinostat achieved IC₅₀ values of 0.14 ± 0.02 µM (with briciclib) and 0.12 ± 0.03 µM (with thiocolchicine), with efficacies of 90.59 ± 12.80% and 94.24 ± 13.74%, respectively. These corresponded to 69.5-fold and 81.1-fold potency improvements over panobinostat alone (IC₅₀ = 9.73 ± 13.40 µM). Additional example curves and hit summaries are provided in Supplementary Fig. S1 and Supplementary Table S6. For example, panobinostat combined with ELR-510444 achieved an IC₅₀ of 0.96 ± 1.54 µM (efficacy = 106.07 ± 14.72%), indicating a 10.1-fold improvement over panobinostat alone. CUDC-907 combined with briciclib, thiocolchicine, or ELR-510,444 yielded IC₅₀ values of 0.07 ± 0.04 µM, 0.10 ± 0.05 µM, and 0.17 ± 0.07 µM (efficacies = 103.19 ± 3.80%, 115.27 ± 9.86%, and 114.88 ± 7.56%, respectively), representing 41.6-fold, 29.1-fold, and 17.1-fold improvements over CUDC-907 alone (IC₅₀ = 2.91 ± 3.04 µM). Brefeldin A combined with DMEP, briciclib, or nocodazole achieved IC₅₀ values of 0.16 ± 0.02 µM, 0.17 ± 0.10 µM, and 0.18 ± 0.06 µM (efficacies = 56.52 ± 14.14%, 63.42 ± 26.44%, and 66.50 ± 17.08%, respectively), corresponding to 12.7-fold, 11.9-fold, and 11.3-fold potency enhancements relative to brefeldin A alone (IC₅₀ = 2.03 ± 2.10 µM). Efficacy values exceeding 100% reflect variability in normalized readouts and curve fitting.

### Matrix-based combination screening reveals pronounced context-dependent synergy

**Fig. 5:**
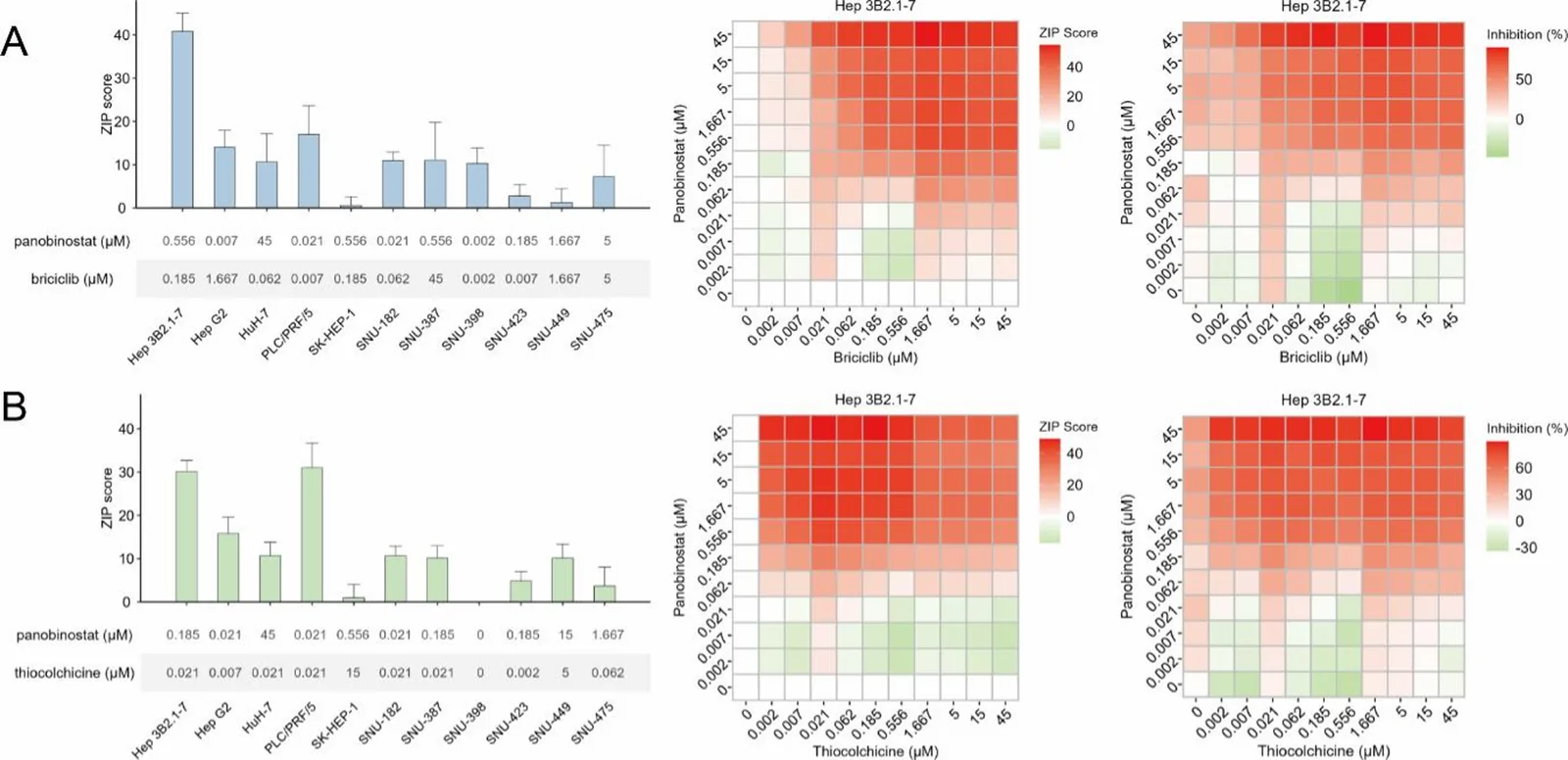
Matrix-based dose-response interaction profiling across 11 liver cancer cell lines. (**A**) Panobinostat plus briciclib. (**B**) Panobinostat plus thiocolchicine. Left panels: ZIP synergy scores for the representative optimal synergy dose pair in each cell line, defined as the dose pair with the lowest sum of concentrations that achieved > 50% viability reduction and ZIP > 10. If multiple pairs met this criterion, the pair with the highest ZIP score was selected. Cell lines without bars had no dose pair meeting this dual criterion. Data are presented as mean ± standard deviation (SD), with error bars representing the SD of two independent experiments. Middle panels: ZIP synergy score maps for the Hep 3B2.1-7 cell line across all 121 dose pairs. This corresponds to 10 non-zero concentrations plus a 0 µM control per compound (11 levels), producing 11 × 11 = 121 dose pairs. Right panels: Percent viability reduction maps for Hep 3B2.1-7 under the same concentration conditions

Dose-response matrices were generated to evaluate the combination effects of panobinostat with either briciclib or thiocolchicine across all 11 cell lines. These two representative combinations were selected for matrix profiling based on (i) the magnitude and reproducibility of potency-enhancement across independent experiments and (ii) mechanistic diversity and translational relevance. In total, 2,662 dose pairs were tested per experiment (121 dose pairs × 11 cell lines × 2 combinations), with each matrix measured in two independent biological experiments (Supplementary Table S7). Across the panel, combination outcomes varied substantially depending on cell type and compound (Table 1). For panobinostat-briciclib, Hep 3B2.1-7 showed the highest fraction of synergistic dose pairs under the dual criterion (ZIP > 10 and inhibition > 50%) (32.2%). In contrast, synergy was ≤ 6.6% in other lines, including PLC/PRF/5 (6.6%), Hep G2 (5.8%), HuH-7 (5.0%), SNU-182 (5.0%), SNU-387 (3.3%), and SNU-398 (0.8%), while SNU-449, SNU-475, SK-HEP-1, and SNU-423 showed no synergistic dose pairs. Antagonism (ZIP < − 10) was most prevalent in HuH-7 (62.0%) and SNU-387 (61.2%). For panobinostat-thiocolchicine, Hep 3B2.1-7 again exhibited the largest synergistic fraction (41.3%), followed by SNU-387 (24.0%) and Hep G2 (12.4%). Lower synergy was observed in PLC/PRF/5 (5.8%), HuH-7 (5.0%), SNU-182 (3.3%), and SNU-449 (1.7%), whereas SNU-398, SNU-475, SK-HEP-1, and SNU-423 showed no synergistic dose pairs. Antagonism was highest in HuH-7 (53.7%), SNU-398 (24.0%), and SNU-423 (24.0%). To summarize combination-specific optimal dose pairings within each cell line, a representative synergy dose pair was defined as the dose pair with the lowest sum of concentrations that achieved > 50% viability reduction and ZIP > 10. If multiple pairs met this criterion, the pair with the highest ZIP score was selected. This criterion favors lower combined exposure while maintaining a phenotypically meaningful effect (Fig. 5, and Supplementary Table S7). For panobinostat-briciclib, the strongest representative synergy occurred in Hep 3B2.1-7, where 0.556 µM panobinostat + 0.185 µM briciclib produced 58.53 ± 6.54% inhibition with ZIP = 40.80 ± 4.22 (mean ± SD; *n* = 2). Representative synergy at substantially lower concentrations was also observed in PLC/PRF/5 (0.021 µM panobinostat + 0.007 µM briciclib, 56.86 ± 0.42% inhibition, and ZIP = 17.02 ± 6.64) and in Hep G2 (0.007 µM panobinostat + 1.667 µM briciclib, 51.91 ± 2.96% inhibition, and ZIP = 14.05 ± 3.90). Other lines yielded representative ZIP scores above 10 with more variable response levels (e.g., SNU-387, SNU-182, HuH-7, SNU-398), while four lines (SNU-475, SNU-423, SNU-449, and SK-HEP-1) had no dose pairs meeting the dual criterion. For panobinostat-thiocolchicine, pronounced representative synergy was observed at low micromolar or submicromolar concentrations in selected models. In PLC/PRF/5, 0.021 µM thiocolchicine + 0.020 µM panobinostat yielded 67.78 ± 25.65% inhibition with ZIP = 31.03 ± 5.67, and in Hep 3B2.1-7, 0.19 µM panobinostat + 0.021 µM thiocolchicine produced 50.28 ± 0.48% inhibition with ZIP = 30.19 ± 2.51. Other lines showed lower synergy (e.g., Hep G2 with ZIP = 15.82 ± 3.75) or marginal effects with ZIP values near 10 (HuH-7, SNU-182, SNU-387, and SNU-449), while SNU-423, SNU-475, SK-HEP-1, and SNU-398 showed no synergy.

**Table 1:** Summary of compound combination interaction profiles across 11 liver cancer cell lines

Cell Line	panobinostat-briciclib			panobinostat-thiocolchicine		
	Synergy	non-synergistic/non-antagonistic	Antagonism	Synergy	non-synergistic/non-antagonistic	Antagonism
Hep 3B2.1-7	39 (32.2%)	78 (64.5%)	4 (3.3%)	50 (41.3%)	60 (49.6%)	11 (9.1%)
PLC/PRF/5	8 (6.6%)	113 (93.4%)	0 (0.0%)	7 (5.8%)	114 (94.2%)	0 (0.0%)
Hep G2	7 (5.8%)	111 (91.7%)	3 (2.5%)	15 (12.4%)	106 (87.6%)	0 (0.0%)
HuH-7	6 (5.0%)	40 (33.1%)	75 (62.0%)	6 (5.0%)	50 (41.3%)	65 (53.7%)
SNU-182	6 (5.0%)	108 (89.3%)	7 (5.8%)	4 (3.3%)	116 (95.9%)	1 (0.8%)
SNU-387	4 (3.3%)	43 (35.5%)	74 (61.2%)	29 (24.0%)	91 (75.2%)	1 (0.8%)
SNU-398	1 (0.8%)	91 (75.2%)	29 (24.0%)	0 (0.0%)	92 (76.0%)	29 (24.0%)
SNU-449	0 (0.0%)	92 (76.0%)	29 (24.0%)	2 (1.7%)	94 (77.7%)	25 (20.7%)
SNU-475	0 (0.0%)	92 (76.0%)	29 (24.0%)	0 (0.0%)	113 (93.4%)	8 (6.6%)
SK-HEP-1	0 (0.0%)	97 (80.2%)	24 (19.8%)	0 (0.0%)	115 (95.0%)	6 (5.0%)
SNU-423	0 (0.0%)	103 (85.1%)	18 (14.9%)	0 (0.0%)	92 (76.0%)	29 (24.0%)

Note: Each combination was evaluated using an 11 × 11 dose-response matrix, corresponding to 121 dose pairs per cell line, in two independent biological experiments. For each dose pair, ZIP synergy scores and percent viability reduction were averaged across the two replicates and then classified using predefined thresholds: synergy (ZIP > 10 and > 50% inhibition), antagonism (ZIP < − 10), and non-synergistic/non-antagonistic (all remaining dose pairs, including ZIP > 10 with ≤ 50% inhibition). Values are shown as number of dose pairs (percentage of 121)

## Discussion

Combination therapy is a key strategy to overcome drug resistance and enhance therapeutic efficacy in cancer. However, systematic identification of synergistic drug combinations remains challenging because of combinatorial scale and biological context dependence. In this study, cell viability profiles of 244 compounds across 11 liver cancer cell lines (9 HCC lines plus two non-HCC liver cancer models) were integrated with bioactivity signatures derived from a diverse panel of 1,925 biochemical and cell-based assays. Using compound activity profile correlation analysis, candidate combinations were systematically prioritized and experimentally evaluated for potential anti–liver cancer activity. These findings provide context-specific preclinical leads for combination strategies in liver cancer and demonstrate the value of integrating orthogonal single-agent screening resources to guide rational combination discovery.

A diverse bioactive compound library was screened in this study (Supplementary Table S2). Structurally, the library spanned simple aromatic scaffolds (e.g., niclosamide) [32] and complex macrocyclic natural products (e.g., epothilone A) [32], covering a wide physicochemical range from highly lipophilic compounds (e.g., paclitaxel) [33] to more hydrophilic molecules (e.g., bortezomib) [34]. Mechanistically, it comprised agents targeting diverse pathways, including microtubule stabilization (e.g., paclitaxel) [35], topoisomerase I inhibition (e.g., camptothecin) [36], proteasome inhibition (e.g., bortezomib) [37], and histone deacetylase inhibition (e.g., panobinostat) [38]. Collectively, this breadth supports coverage of key oncogenic processes (e.g., cell-cycle regulation, apoptosis, DNA-damage response, epigenetic modulation, and proteostasis) and facilitates identification of complementary pharmacological interactions. Most compounds were approved drugs or well-characterized bioactives with known mechanisms, while a subset comprised less-studied entities (Supplementary Table S2), enabling both repurposing opportunities and discovery of novel combination hypotheses for liver cancer therapy.

Phenotypic drug discovery has emerged as a powerful strategy for addressing complex, poorly understood diseases, and has delivered first-in-class drugs with novel mechanisms of action [24, 39]. In line with this paradigm, a molecularly diverse panel of 11 liver cancer cell lines was assembled to reflect clinically relevant HCC heterogeneity, with emphasis on *TP53* alteration patterns (Supplementary Table S1). The panel included a *TP53*-null cell line with complete loss of p53 expression (Hep 3B2.1-7 [40]), one line with a nonsense mutation (SNU-387), six lines with missense mutations (PLC/PRF/5, HuH-7, SNU-182, SNU-449, SNU-475, and SNU-398), and one line with a splice-site alteration (SNU-423) [41]. These cell lines were used to generate phenotypic cell viability profiles. Compound activity was summarized using a composite score integrating potency and efficacy (Fig. 2A and Supplementary Table S3), providing a more informative prioritization metric than IC₅₀ alone [42]. In parallel, target-based screening offers mechanistic resolution and enables large-scale interrogation of cancer-relevant targets [25, 43]. The 1,925 biochemical and cell-based assays used here spanned major target classes, including kinases (e.g., epidermal growth factor receptor (EGFR), and Bruton’s tyrosine kinase (BTK)), nuclear receptors (e.g., androgen receptor (AR), and estrogen receptor (ER)), DNA damage response components (e.g., poly(ADP-ribose) polymerase (PARP), and ataxia telangiectasia and Rad3-related (ATR) kinase), and metabolic enzymes such as cytochrome P450 (CYP) isoforms (Fig. 3 and Supplementary Table S4). These target classes have driven important therapeutic advances in oncology, such as EGFR inhibitors in *EGFR*-mutant lung cancer [44], BTK inhibitors in B-cell malignancies [45], PARP inhibitors in *BRCA1/2*-deficient cancers [46], and WEE1/ATR inhibitors in *TP53*-defective tumors [47].

Phenotypic and target-based screening are often presented as competing paradigms for drug discovery, yet their integration can leverage complementary strengths [48, 49]. Phenotypic screens capture integrated cellular responses but typically provide limited mechanistic resolution, whereas target-based assays offer molecular insight yet may not translate to cellular activity because of permeability, efflux, and/or pathway redundancy [39]. For example, the development of PARP inhibitors illustrates how linking phenotype to mechanism can yield transformative therapies [50]. The 2005 demonstration that PARP inhibition selectively kills BRCA1/2-deficient cells via synthetic lethality provided both phenotypic validation and mechanistic rationale, ultimately leading to olaparib approval for BRCA-mutated ovarian cancer. A key feature of this work is the systematic integration of phenotypic and target-based data at the compound-compound level (Fig. 1). Correlation coefficients were computed for compound activity profiles across (i) 11 liver cancer cell lines and (ii) 1,925 biochemical and cell-based assays, followed by stringent dual filtering in both datasets. This reduced the combination candidate space to 821 high-confidence pairs involving 190 unique compounds (~ 36-fold compression) (Supplementary Table S5). Compounds were ranked by their frequency of occurrence within retained pairs, enabling selection of eight anchor compounds within 125 library compounds (992 anchor–library pairs) for fixed-anchor-concentration experimental validation (Fig. 1).

The fixed-anchor-concentration primary screen identified 89 combinations with marked potency enhancement (Supplementary Table S6). Among these, panobinostat combined with either briciclib or thiocolchicine produced pronounced potency-shifts in the Hep 3B2.1-7 cell line (Fig. 4), which were advanced to follow-up with 11 × 11 dose-response matrix screen across the full cell-line panel (Fig. 5 and Supplementary Table S7). Mechanistically, these observations are consistent with a stress-integration hypothesis. Panobinostat, a pan-histone deacetylase (HDAC) inhibitor, remodels acetylation-dependent transcription and proteostasis programs [38, 51], and modulates non-histone substrates involved in cytoskeletal regulation [52] and stress responses [53]. For the panobinostat-briciclib combination, briciclib is a styryl-sulfone-related compound that has been reported to perturb cell-cycle regulators (including cyclin D) in cancer models, providing a rationale for combining cell-cycle/translation stress with the epigenetic stress induced by HDAC inhibition [54, 55]. For the panobinostat-thiocolchicine combination, thiocolchicine is a microtubule-disrupting agent that imposes mitotic and proteotoxic stress, and HDAC inhibition has been reported to sensitize tumor models to mitotic perturbation in multiple settings [56, 57]. These mechanistic links are hypothesis-generating because the readout in this study measures cell viability as a phenotype rather than pathway-resolved target engagement.

The dose–response matrix screen delineated combination-specific dose windows while revealing strong context-dependent variations relevant for translational decision-making. Regions of high inhibition coinciding with strong positive ZIP scores identified synergistic dose combinations that may be actionable for further development. For example, in Hep 3B2.1-7, 0.556 µM panobinostat + 0.185 µM briciclib yielded 58.53 ± 6.54% inhibition with ZIP = 40.80 ± 4.22, while in PLC/PRF/5, 0.020 µM panobinostat + 0.021 µM thiocolchicine achieved 67.78 ± 25.65% inhibition with ZIP = 31.03 ± 5.67 (Fig. 5, and Supplementary Table S7; mean ± SD from *n* = 2 independent experiments). Such windows motivate pharmacokinetic/pharmacodynamic (PK/PD)-aligned studies to determine whether synergistic concentrations are achievable and tolerable in vivo. Conversely, extensive antagonism in some contexts (Table 1) underscores the need to evaluate combinations across relevant cell models and dose regions to avoid advancing pairs into counterproductive settings. These results also illustrate the limits of single-feature stratification. Although strong synergy in the TP53-null Hep 3B2.1-7 cell line is consistent with the hypothesis that p53-pathway dysfunction may modulate combination sensitivity in specific contexts [58], responses diverged across other TP53-altered lines. For example, panobinostat-thiocolchicine showed predominant antagonism in HuH-7 but minimal antagonism in SNU-387 (Table 1), suggesting that TP53 status alone is insufficient to predict combination response in HCC [59]. More informative stratification will likely require integration of TP53 mutation class, co-occurring pathway states, epigenetic features, mitotic control, stress-response capacity, and protein-turnover/neddylation activity. In addition, the extreme cases, such as PLC/PRF/5 with no antagonistic dose pairs (0/121) versus SK-HEP-1 with no synergistic dose pairs (0/121) for panobinostat-thiocolchicine (Table 1), motivate targeted molecular characterization (e.g., epigenetic state [60], mitotic control [61], and stress response circuitry [62]) to define determinants of favorable versus unfavorable combination landscapes.

Our correlation-based prioritization strategy is complementary to supervised machine-learning approaches for synergy prediction. Supervised models can integrate chemical structure, genomic features, and prior combination outcomes to make context-specific predictions [20, 21], but they rely on large annotated synergy datasets that remain limited for HCC-relevant settings [63]. In contrast, the label-free approach used here leverages existing single-agent profiling resources and does not require pre-existing combination outcome labels. The working hypothesis is that compounds exhibiting concordant activity patterns across a diverse panel of phenotypic and target-based assays may be enriched for pharmacological interactions that can manifest as synergy, antagonism, or additivity depending on cellular context. Future studies integrating assay-level pathway annotations, transcriptomic or proteomic profiling, and functional rescue experiments will be needed to determine whether synergistic dose pairs converge on HDAC-regulated transcription, mitotic stress, proteostasis, apoptosis, or other HCC-relevant pathways.

This study has several limitations. First, we quantified only a single endpoint, cell viability, at one time point in 2D monolayer cultures, which cannot resolve temporal response dynamics, distinguish cytostatic from cytotoxic effects, or capture tumor-microenvironmental and immune contributions [64]. Future studies should incorporate time-resolved live-cell imaging, apoptosis and cell-cycle markers, and clonogenic recovery assays to define whether the observed viability reduction reflects durable cytotoxicity or reversible growth arrest. Translational interpretation will also require validation in higher-fidelity systems, including 3D spheroids, patient-derived organoids, and in vivo models, together with PK/PD and tolerability assessments, particularly under altered hepatic metabolism [65]. Second, this study did not include normal hepatic models, and therefore the therapeutic selectivity of these identified combinations (panobinostat-briciclib and panobinostat-thiocolchicine) cannot be determined from the present data. Previous studies on these drugs tested as single agents in non-tumorigenic cell models can provide some context on the relative safety of these compounds: panobinostat did not significantly reduce viability in the non-tumorigenic human liver cell line LO-2 at 25–100 nM [66], briciclib showed limited toxicity in normal endothelial cells (liver-specific toxicity data for briciclib are unavailable) [67], and thiocolchicine maintained approximately 91–94% viability in primary human hepatocytes at 1-100 µM [68]. These single-agent observations do not establish combination selectivity, and future studies should evaluate these identified combinations directly in normal hepatic models and calculate tumor-versus-normal selectivity indices to define the therapeutic window. In addition, the matrix-based synergy experiments were performed in two independent biological replicates, which limits the statistical power of individual ZIP estimates. Therefore, the reported synergy classifications and representative dose pairs should be interpreted as prioritized leads rather than definitive synergy calls. The most actionable combinations warrant confirmation using additional biological replicates, orthogonal synergy models, and higher-fidelity experimental systems.

Future work should define biomarkers and mechanisms underlying the context-dependent synergy and antagonism observed here. Broader oncology studies linking vascular endothelial growth factor-A (VEGF-A) expression in acute lymphoblastic leukemia (ALL), pharmacogenomic variants in multiple myeloma (MM), lactate-related signatures in laryngeal squamous cell carcinoma (LSCC), and neddylation-related signatures in cutaneous melanoma (CM) to prognosis or treatment response support future HCC-specific biomarker and pharmacogenomic stratification [70–73]. Mechanistic follow-up should include apoptosis-related readouts such as BCL2-associated X, apoptosis regulator (BAX), B-cell lymphoma 2 (BCL-2), and caspase-3, as well as cytokine/TME perturbation assays to determine whether the prioritized combinations engage cell-death and microenvironment-regulated response programs [74, 75]. Computational extensions, including molecular dynamics (MD)/virtual reality (VR) visualization, generative artificial intelligence (AI)-guided molecular design, docking-based hypothesis generation, and cell-physiology-informed modeling, may further support experimentally testable hypotheses for HCC validation [76–79].

## Conclusion

We applied correlation analysis to compound activity profiles generated across a large, diverse panel of phenotypic and target-based assays to prioritize candidate compound combinations for experimental testing in liver cancer cell lines. This correlation-based approach substantially reduced the combinatorial search space and yielded a focused set of candidate combinations for experimental confirmation and follow-up studies. Two combinations, panobinostat–briciclib and panobinostat–thiocolchicine, exhibited dose-dependent and context-specific interaction patterns in matrix-based assays, underscoring both therapeutic potential and the heterogeneity of combination responses across liver cancer models.

These findings provide hypothesis-generating, context-specific preclinical leads for further evaluation in higher-fidelity systems and offer a generalizable template for data-driven combination discovery in molecularly heterogeneous malignancies.

## Supplementary Information

Below is the link to the electronic supplementary material.

Supplementary Material 1

## Data Availability

All processed data underlying the figures and tables are provided in Supplementary Tables S1–S7. The qHTS dataset reported in this paper is available in PubChem (AID 2202548).
